# Post-Traumatic Stress Reactions in Caregivers of Children and Adolescents/Young Adults with Severe Diseases: A Systematic Review of Risk and Protective Factors

**DOI:** 10.3390/ijerph18010189

**Published:** 2020-12-29

**Authors:** Claudia Carmassi, Valerio Dell’Oste, Claudia Foghi, Carlo Antonio Bertelloni, Eugenia Conti, Sara Calderoni, Roberta Battini, Liliana Dell’Osso

**Affiliations:** 1 Department of Clinical and Experimental Medicine, University of Pisa, 56126 Pisa, Italy; claudia.carmassi@unipi.it (C.C.); foghi.claudia@gmail.com (C.F.); carlo.ab@hotmail.it (C.A.B.); scalderoni@fsm.unipi.it (S.C.); rbattini@fsm.unipi.it (R.B.); liliana.dellosso@med.unipi.it (L.D.); 2 Department of Biotechnology, Chemistry and Pharmacy, University of Siena, 53100 Siena, Italy; 3 IRCCS Stella Maris Foundation, 56128 Pisa, Italy; econti@fsm.unipi.it

**Keywords:** post-traumatic stress disorder (PTSD), post-traumatic stress symptoms (PTSS), caregiving burden, parents, psychological distress

## Abstract

Severe illnesses in children and adolescents/young adults (AYAs) may represent a complex burden for patients and their caregivers, including a wide range of mental disorders, particularly post-traumatic stress disorder (PTSD). Few events are as potentially traumatizing as having a son or a daughter diagnosed with a severe, life-threatening, or disabling disease. The presence of PTSD symptoms in caregivers may compromise their efficacy as caregivers and negatively affect the child’s well-being. This systematic review aims at outlining potential risk and protective factors for the development of PTSD symptoms in caregivers of children and AYAs affected by severe acute or chronic illnesses. Thirty-one studies on caregivers of children and AYAs affected by severe, acute, or chronic diseases were included. Socio-demographic and socio-economic characteristics, illness-related distress, psychiatric symptoms, support, and coping styles were found as potential risk/protective factors across studies. It is crucial to consider risk factors affecting caregivers of severely ill young patients, in order to plan focused interventions aimed at preventing an adverse clinical outcome in caregivers and at enhancing caregivers’ coping skills, in order to ultimately improve their quality of life.

## 1. Introduction

Severe acute or chronic illnesses represent a stressful burden for patients and their caregivers. Moreover, they may favor the onset of a wide range of psychological problems in carers, such as depression, anxiety, sleep disturbances, and post-traumatic stress disorder (PTSD) [1,2,3,4,5,6,7,8,9]. PTSD is a condition that may occur after traumatic events, life-threatening events, or painful experiences [10,11,12]. Subjects with PTSD are not necessarily the victims of an event: witnessing, being involved, or being informed of a traumatic event happened to a beloved one may trigger the onset of PTSD [13]. In this regards, few events are as potentially traumatizing as having a child diagnosed with a possibly life-threatening or disabling disease [14]. Particularly, the Diagnostic and Statistical Manual of Mental Disorders—fifth edition (DSM-5) introduced illness in one’s child among the events considered as traumatic (“a medical catastrophe concerning one’s child”), specifying that the event must represent a condition that endangers life at the moment [15]. Consequently, this definition overcame the previous DSM-IV-TR statement that required learning that one’s child has a life-threatening disease [16,17], highlighting the need for the traumatic event to be sudden and dangerous, focusing on the urgency and abruptness of the perceived threat [18,19]. However, many authors have also discussed the clinical relevance of so-called “minor” events in the development of full and, similarly disabling, partial post-traumatic symptomatology, adopting a wide approach of spectrum to trauma and traumatic event, focused on individual experience vulnerability [20]. In this perspective, increasing attention has been deserved to PTSD among parents and caregivers of paediatric patients affected by severe but heterogeneous medical conditions characterized by a severe impairment or a chronic course, even a significant risk of mortality, as cancer, leukaemia/lymphoma, diabetes, neurological acute (ischemic, haemorrhagic, traumatic injury) or chronic (epilepsy) conditions, psychiatric and neurodevelopment disorders (such as autism), sex development disorders, burn injury, severe asthma crisis, illnesses that needed transplant surgery, pointing out the potential traumatic role of such experiences [21,22,23,24]. Severe medical conditions occurring to loved ones could be one of the strongest factors associated to PTSD [24]. However, only recently researchers began to focus on PTSD in parents of children with chronic illnesses [14,21,22,23]. Studies using the DSM criteria for PTSD diagnosis reported incidences of current cancer-related PTSD ranging from 6.2% to 25% parents’ childhood cancer survivors, while clinically severe levels of PTSD symptoms ranged from 9.8% to 44% [21]. Similarly, in diabetes type 1 studies, up to a quarter of the parents met the DSM criteria for PTSD diagnosis 6 weeks after their child’s diagnosis, while 15% of mothers still met criteria for a partial diagnosis of PTSD and 10% met criteria for the full diagnosis up to 5 years after [23].

Post-traumatic stress symptoms (PTSS) include nuanced or attenuated symptomatology belonging to the traumatic reaction, as intrusion symptoms, persistent avoidance, negative alterations in cognitions and mood, alterations in arousal and activity. Nevertheless, growing findings suggest partial or sub-threshold PTSD conditions being related to an impairment in family, work, and social adjustment as full-blown syndromes, often representing a problem not only during the acute phase but also across extended periods after the traumatic exposure [25,26]. PTSS in parents may affect their caregiver role and adversely impact on the child’s behavior, response to treatment, and treatment outcome. Arousal or re-experiencing symptoms, in fact, may compromise the caregiver’s adherence to medical guidelines or to report essential information to health professionals [27]. Hyper-vigilant caregivers may request frequent doctor visits or make an excessive number of accesses to medical services [28]. On the other hand, caregivers with active avoidance may pass over medical visits or refuse necessary medical procedures [29]. Parallel, the impact of parent’s stress on the child may be detrimental for the child’s well-being [30], and PTSD symptoms in parents can have negative implications for their child’s behavior or adjustment in the long-term, as well as impair the parent–child relationship [31]. Moreover, while struggling with their difficulties in dealing with stress, caregivers often have a reduced access to support and resources [32,33]. Furthermore, PTSD has been positively associated to an increased risk of other psychopathological conditions and substance abuse, leading to a well-being impairment [34,35,36]. In this line, increasing evidence suggests that PTSD or PTSS could be associated to the onset of depression, suicidal behavior, anxiety, sleep problems, and other conditions in cares that worse their quality of life [1,2,4,7,9].

Although a lack of studies in this research area [37], interventions to prevent PTSS, and enhance resilience are likely to relieve the suffering of the parents (and indirectly of the young patients) and maintain their caregiver ability. Indeed, some studies found a role of resilience-focused interventions in improving caregivers’ quality of life [38,39]. It seems primary to recognize possible PTSD risk factors (such as gender, age, carers’ mental health history, characteristics of the child’s illness, and other factor that could be positively associated to PTSD), as well as other variables that could represent protective factors for PTSD development in cares (as positive coping styles or availability of familiar of healthcare service support, and other variables which could be negatively associated to PTSD). Therefore, the present systematic review aimed at exploring the possible risk and protective factors for the development of PTSD and PTSS in caregivers of children and adolescents/young adults (AYAs) affected by severe illnesses, in order to plan interventions aimed at promoting the mental well-being of both caregivers and young patients.

## 2. Materials and Methods

### 2.1. Literature Search

A systematic search was conducted from 1st September 2020 to 30th September 2020 by searching the electronic databases PubMed, Ovid, and EMBASE. All studies from 1 January 1990 to 31 August 2020 were included in the databases search. The combination of search terms without filters, restrictions, or limits was utilized: (PTSD OR post-traumatic stress OR post-traumatic stress disorder OR post-traumatic stress symptoms OR PTSS OR post-traumatic stress reaction) AND (caregiver OR caregivers OR parents OR mothers OR fathers).

### 2.2. Eligibility Criteria

The following criteria were utilized to include studies in the present review:

1. Full-text publications reporting data related to PTSD and PTSS in caregivers of children and AYAs (aged between 0–25 years) affected by severe illnesses (i.e., chronic or acute, life threatening or disabling diseases).
2. Articles assessing possible risk (positively associated) and/or protective (negatively associated) factors for PTSD and PTSS in caregivers.
3. Articles available in English.

### 2.3. Screening and Selection Process

**Figure 1:**
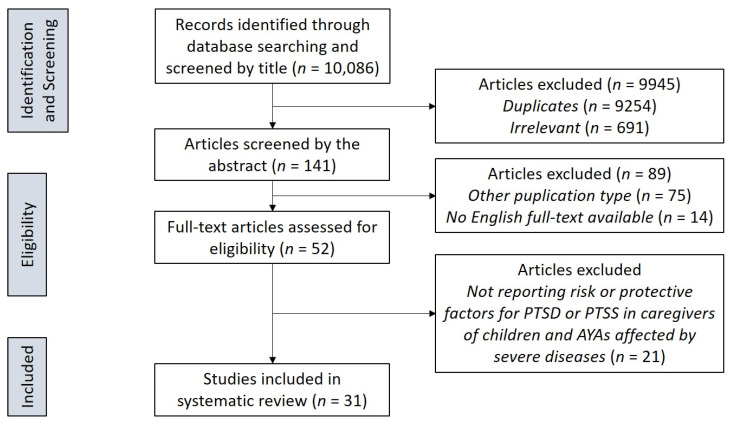
PRISMA (Preferred Reporting Items for Systematic reviews and Meta-Analyses) flowchart of the study selection process.

The primary database search provided a total of 10,086 records. Whereupon, 10,034 publications were removed after titles and abstracts screening because they were duplicates (*N* = 9254) or not relevant (i.e., they did not investigate the PTSD symptoms, or caregivers or were not research studies) (*N* = 691), or because full-text was not available or not in English (*N* = 14), or were reviews or metanalyses (*N* = 75). Fifty-two articles passed the initial screening. The relevant references cited in the selected studies, besides in review and metanalyses found in the initial search, were also manually screened to complete our search. However, no further eligible studies emerged in the process of manual screening. Data extraction and eligibility assessment were performed independently by two raters. Any disagreements highlighted during the process was discussed, and consensus reached. Finally, 31 studies were included in the present review. Details of the screening process are summarized in Figure 1, illustrating a flow chart according to PRISMA (Preferred Reporting Items for Systematic reviews and Meta-Analyses) recommendations [40].

## 3. Results

The search yielded 31 studies that were included in the review (see Figure 1). In our search, the large majority of the studies analyzed was on caregivers of children and AYAs affected by tumors, investigated by fourteen studies [41,42,43,44,45,46,47,48,49,50,51,52,53,54]. The remaining diagnostic categories of children and AYAs whose caregivers were investigated for PTSD were very heterogeneous and less numerous, and included neonatal intensive care unit (NICU) patients [55,56], epilepsy [57,58], burn injuries, and other accidents [41,59,60,61], disorders of sex development [62,63], asthma [64], transplants [65,66,67,68], HIV [66], Sickle cell disease [66], diabetes mellitus type 1 [41], and neuro-psychiatric disorders [69,70,71]. Characteristics of included studies are described in Table 1.

A large majority of the studies adopted the Impact of Event Scale- Revised (IES-R) [42,43,44,45,48,53,54,55,61,62,63,66,67,69], a self-report questionnaire used to assess probable PTSD by covering all three symptoms clusters (intrusion, avoidance, hyperarousal) through 22 items and in which individuals with a score equal or above 24 were considered to have clinically significant post-traumatic stress symptoms (PTSS), while those with a score equal or higher than 33 a probable PTSD diagnosis [72]. Other tools used in the studies included in the present review were various version of the Posttraumatic Stress Disorder Checklist (PCL) [47,49,50,51,52,59,71]. Particularly, the PCL, the PCL-civilian (PCL-C) and the PCL- specific (PCL-S) are self-report rating scale for assessing the 17 DSM-IV symptoms of PTSD. The cut-off for assessing PTSD diagnosis among studies. The PCL for the DSM-5 (PCL-5) is a 20-item self-report measure that assesses the 20 DSM-5 symptoms of PTSD. A cut-off of >33 combined to the endorsement of a cluster criteria represents a good predictor of a PTSD diagnosis [73,74]. Moreover, with this scale PTSD symptoms were identified as clinically significant (PTSS) if the participant was at least “moderately bothered” by at least one reexperiencing cluster symptom, three avoidance cluster symptoms, or two arousal cluster symptoms [59]. Four studies used the post-traumatic diagnostic scale (PDS), a 49 item self-report questionnaire corresponding to the DSM-IV PTSD criteria and in which individuals received a probable PTSD diagnosis if their answers met DSM-IV PTSD Criteria A-F, while a score equal or higher than 21 was considered predictive of at-least moderate-severe PTSS [60]. Two studies used the trauma and loss spectrum-self report (TALS), a self-report tool including 116 items that explores the lifetime experience of losses, traumatic events and PTSD symptoms, behaviors and personal characteristics that might represent manifestations and/or risk factors for the development of PTSD [26]. With this instrument, DSM-5 PTSD diagnosis was assessed utilizing a matching system between symptom criteria and TALS-SR items, and a probable partial PTDS diagnosis was predicted if individuals met two or three of the DSM-5 B, C, D, and E criteria for PTSD [57,58]. Finally, the remaining tools were used only once, and two were self-report measures: the Post-traumatic Stress Disorder Assessment Scale [46], the Post-traumatic stress disorder questionnaire (PPQ) [56], and the Clinician-Administered PTSD Scale (CAPS) [68], a questionnaire administered by a trained clinician.

Particularly, PTSD prevalence rates ranged from 4.44% [59] to 82.9% [55], and PTSS prevalence rates from 3.9% [48] to 54% [67].

### 3.1. Risk Factors for PTSD/PTSS

We identified the following risk factors for PTSD and PTSS: socio-demographic and socio-economic characteristics; illness-related distress; psychiatric symptoms and negative/maladaptive coping.

#### 3.1.1. Socio-Demographic and Socio-Economic Characteristics

Women were found to be at statistically significantly higher risk of developing PTSS with respect to men in five studies [42,46,50,58,63]. In a sample of 134 parents of pediatric patients with epilepsy, Carmassi et al. reported PTSD in 13.3% of mothers and 4.5% of fathers, and an additional 43.3% of the mothers and 25.0% of the fathers, presented partial PTSD [58]. Younger age of one’s child/AYA was found to be a risk factor for developing PTSD in caregivers in four studies [51,59,61,62]. Moreover, other authors found that younger age of parents too was related to an increased risk of PTSD [50]. Furthermore, some authors [49], examining a sample of 204 parent caregivers of AYAs with cancer, found that the presence of other life stressors, a more severe impact of patient’s illness on plans for the future and on the broader family, was associated with increased PTSS levels. These authors also highlighted an association between PTSS and living outside the metropolitan area. A study on a sample of 265 parents of children with autism spectrum disorder (ASD) showed that having other life stressors, as having one biological parent not living in the home, having two or more children diagnosed with ASD, difficulties finding or holding a job or marital problems, were related to an increased risk of PTSS [69]. Moreover, having a lower household income and increased expenses related to the disease, were found to be associated to PTSD symptoms severity in fathers [53,63]. More widely, some other authors investigating a sample of 45 parents of children post-burn, found that the level of familiar stress prior to the burn event was related to higher PTSS in caregivers [59]. Finally, only one study pointed out that married subjects had an increased risk of developing PTSS [67].

#### 3.1.2. Illness-Related Distress

Longer stays in hospital [61] as well as objective illness severity [56] have been associated to increased risk of PTSD. Ingerski et al. [66], in a sample of 64 caregivers of children experiencing a chronic illness, found that being caregivers of transplant candidates was a risk factor for developing PTSD. Moreover, another study on 215 caregivers of children undergoing hematopoietic stem cell transplant, highlighted higher PTSS levels in caregivers of allogeneic transplant patients, a procedure associated with greater medical complications and risk of mortality than autologous transplant [67]. Perez et al. [53], in a study on 59 parents of children with cancer, found that illness uncertainty acted indirectly as a risk factor, through ruminations, in enhancing PTSS. Moreover, in a more recent study on the same sample expanded up to 145 caregivers of children with cancer, authors found a significant direct relationship between illness uncertainty and PTSS [54]. The disease status was also found to be an important factor, as parents of relapsed children [51] and parents of patients with a greater number of tumor recurrences [44] were at higher risk of PTSS, as well as parents of recently diagnosed patients [42]. Conversely, being no longer on active treatment was associated to a reduction in PTSS [43]. De Young et al. [60], in a sample of 120 parents of children with burn injuries, found that having a higher number of invasive procedures was related to higher PTSS. Another study found an association between objective severity of disease and PTSS. Indeed, Landolt et al. [41] found in a sample of 355 parents of children 5–6 weeks after an accident or a new diagnosis of cancer or diabetes mellitus type 1, that having a diagnosis of cancer were related to higher PTSS in caregivers with respect to the other diagnosis or a poorer functional status of the child. Furthermore, Carmassi et al. [57], in a study on 134 parents of children with epilepsy, highlighted the subjective impact of medical event as a factor enhancing PTSS and, similarly, a greater subjective illness severity was found to be a risk factor by two other studies [47,56]. Indeed, Juth et al. [47] on a sample of 110 caregivers of AYA cancer patients undergoing active treatment at an outpatient clinic, found an association between caregivers’ subjective illness severity and their own PTSS. Malin et al. [56], examining a sample of parents of infants who were in the NICU for more than 14 days, found that parent perceptions of illness were associated with PTSD symptoms, after adjusting for objective measures of illness. As concerns the unknown diagnosis, in another study on a sample of 139 parents of young children with disorders of sex development researchers found that, in fathers, lack of identifiable etiology underlying the disease increased the risk of developing PTSD symptoms [63]. Finally, Stewart et al. [71], investigating a sample of 395 parents of children with ASD and rare diseases, found that challenging behaviors (i.e., persistent and pervasive maladaptive behavior(s)—including physical aggression, self-injurious behaviors, suicidal behaviors, sexually inappropriate behavior, offending behavior such as arson or stealing, elopement, pica—that has a significant adverse effect on the quality of life and/or health and safety of the individual or others) in the child were related to increased risk of PTSD in the caregivers.

#### 3.1.3. Psychiatric Symptoms and Negative/Maladaptive Coping

Psychiatric symptoms, such as depressive and anxiety symptoms [67,69], manic symptoms among fathers [57] and acute distress symptoms [60] were found to be risk factors for PTSS, as well as having a prior trauma history [60] or having a family mental health diagnosis [59]. Other authors found, in a study on 82 parents of infants previously treated in NICU, that PTSS in one partner was related to PTSS in the spouse [55]. Psychiatric symptoms in young patients also resulted as a risk factor for PTSD in parents, as shown by many authors [42,59,60,68]. Indeed, De Young et al. [60] highlighted as child PTSS were associated to higher PTSS in caregivers, and similar findings were shown by Phipps et al. [42] in a sample of 120 parents of children with cancer and by Taskiran et al. [68] in a sample of 27 mothers of children who underwent a bone marrow transplantation. Moreover, Malpert et al. [48], in a sample of 127 parents of long-term survivors of childhood acute lymphoblastic leukemia, found that perceived caregiver strain was significantly associated with PTSS and another study highlighted as, in fathers, the level of perceived stress was related to PTSS [55]. Ruminations as well, resulted to be associated to PTSD symptoms [53] and, in one study, ruminations were found to be associated to higher PTSS in fathers [58]. Negative attitudes and perceptions [65], as well as feelings of guilt and shame [61] and dysfunctional cognitions [70] have shown a correlation with PTSS. Particularly, Young et al. [65] reported, in a sample of 170 caregivers of pediatric transplant recipients, that parents who had more negative attitudes about their health care services and about health care in general were more likely to report more severe PTSD symptoms. Moreover, Pasterski et al. [62] found in a sample of 47 parents of children diagnosed with a disorder of sex development, that having a cognitive response to the diagnosis characterized by confusion and disbelief was associated to an increased risk of PTSD. Another study on 120 caregivers of children with asthma found that following beliefs congruent with a lay model of asthma management, instead of a professional model of disease management, was associated with an increased risk of developing PTSS [64]. Finally, some authors found that some maladaptive coping styles were related to higher PTSS, particularly emotion-oriented coping in fathers and avoidance-oriented coping style in mothers [55].

In conclusion, female gender, a younger age (both of young patients and caregivers), the presence of other stressor and the severity of the illness are the most recognized risk factors for PTSD or PTSS. Furthermore, various kinds of psychiatric symptoms or maladaptive coping strategies are also reported to be associated to PTSD/PTSS.

### 3.2. Protective Factors for PTSD/PTSS

For what concerns the protective factors, we found factors as social status, support, and positive coping emerging across studies.

#### 3.2.1. Social Status

Khalifa et al. [46], in a sample of 96 parents of children with acute lymphoblastic leukemia, reported that higher professional and educational levels were associated with lower PTSS.

#### 3.2.2. Support

Some studies highlighted the importance of support in reducing PTSS. Particularly, reducing barriers to treatment for their child with cancer was found to reduce PTSS [54]. Sawyer et al. [51], on a sample of 204 parents of AYAs with cancer, found that self and child unmet needs of practical support from healthcare services was related to higher PTSS levels. McCarthy et al. [52], analyzing the same study sample, highlighted the important role of meeting the information needs of parents in reducing their PTSS levels.

#### 3.2.3. Positive Coping

Harper et al. [45], examining 75 parents of pediatric cancer patients, found that parents’ caregiving self-efficacy reduced immediate and longer-term PTSD symptoms related to one’s child treatment procedures. Moreover, Bruce et al. [44], in a sample of 52 parents of children with brain tumor, found that having more positive parent–child interactions, as shown by a higher score of “Conflict Resolution”, was related to a reduction in PTSS. Another study on 91 parents and primary caregivers of children recruited in the wards or outpatient clinics during the first 8 weeks following their child’s burn injury, found that parents who rated high in self-compassion reported fewer PTSS [61].

In general, protective factors are less investigated in literature than risk ones. Most of the existent evidence concern positive coping style and support provided by medical staff. Moreover, education levels were negatively associated with PTSS in one study.

## 4. Discussion

The present review summarizes the relevant risk or protective factors for PTSD/PTSS in caregivers of children/AYAs affected by severe, life-threatening, or chronic diseases. We found some risk or protective factors were supported by many studies, while other factors had scant evidence, highlighting the need for further research on this topic. Particularly, although several factors, such as the presence of psychiatric symptoms or support by medical staff could be modified, they are not extensively investigated. In our opinion further studies on these variables are needed to corroborate the previous findings and identify the possible strategies to reduce PTSS.

In regard to sociodemographic factor, our review found women to be at higher risk of developing PTSD symptoms with respect to men [42,46,50,58,63], with rates up to 13.3% of the mothers reporting full-PTSD and 43.3% partial PTSD [58]. This heightened risk of PTSD in women was highlighted in many studies [75,76,77] and has been related to a greater fear conditioning in women than in men [78]. On the other hand, the effect of age on the risk of PTSD/PTSS was investigated with a minor extent. In some studies, younger age of caregivers or patients were a risk factor for PTSD. However, usually studies do not distinguish their results for children, adolescents, or young adults. Moreover, in some cases age range of patients was not clearly reported, hence the effect of age cannot be assessed. Future studies would evaluate if risk factors change in caregivers of young patients with different age, in order to improve specific management strategies. Another major finding was the relevant role of life stressors in increasing parents’ PTSS levels and distress, such as the impact of patient’s illness on plans for the future, the effect of the illness on the broader family [49], and having a lower income while facing the great amount of expenses related to the disease [54,63]. Other authors found that having one biological parent not living in the home, having two or more children diagnosed with ASD, difficulties finding or holding a job or marital problems, were risk factors, reinforcing the role of other life stressors in determining PTSD onset [69]. These results highlight the considerable and complex burden parents may carry out while facing their child’s severe illnesses, and point out the importance of assess all possible stressor and negative life event in such population, in order to detect subjects at risk of PTSD. Considering these data we would suggest a family-centered supportive care for patients and parents, to avoid negative outcomes [79].

Interestingly, we found that illness uncertainty, a cognitive appraisal strongly related to parental distress [80], acted both directly and indirectly, through ruminations, in enhancing PTSS [53,54]. Illness uncertainty mediated the relationship between barriers to care and PTSS, suggesting that greater perceived barriers were related to higher illness uncertainty, which in turn increased parent psychological distress [54]. Moreover, these results suggest that it could be beneficial to the establishment of services aimed at minimizing medical care barriers and consequently at reducing the parental uncertainty [81].

Furthermore, we found that some studies focused on the role of ruminations in developing PTSS [53,58]. Rumination is considered a maladaptive attempt to cope with distress, widely investigated as a potential emotion regulation method, or a possible strategy to cope distressing emotions [82,83]. PTSD symptoms may also been triggered by perseverative thoughts [84,85,86] and, as we found in our review, a significant relationship between PTSS and caregiver rumination emerged [87,88].

Interestingly, feelings of guilt and shame were also found to be associated to higher PTSS in caregivers [60,61]. Feelings of guilt and shame have been recognized by the DSM-5 as having a central role in PTSD psychopathology [15]. Particularly, they were highly reported by patients with PTSD and strongly associated with maladaptive behaviors [89]. Consequently, more recognition should be given to parents’ subjective appraisals of their child’s injury/disease [90]. Moreover, with shame the entire self is seen as at fault, while with guilt the focus is just on specific behaviors [91]. Therefore, shame was found to be a stronger predictor of psychological distress, maybe because it is a more aversive emotional experience compared to guilt [92]. However, as this finding was supported only by two studies, further research should be added on this topic. Guilt and shame feelings were, in fact, related to PTSD development and severity in other populations [25,89] and can also be treated [93], but more specific studies are needed in caregivers.

As concerns protective factors, some studies found the important role of meeting the support needs of caregivers in protecting against PTSD symptoms [51,52,54]. Particularly, access to a social worker for practical support, access to a mental health clinician, and access to an education and vocational advisor for both caregivers and ill young patients have been related to a reduction in the emotional distress of caregivers. These studies also highlighted the important protective role of meeting information needs of caregivers, particularly about the financial impacts of disease for the family. This supports other findings that have reported as families experience significant financial concerns toward the future and have difficulties in accessing income support measures while managing a severe illness in their child [94].

With regard to positive coping styles, some authors [45] found that parents’ caregiving self-efficacy reduced their immediate and longer-term PTSD symptoms related to their children’s treatment procedures. Difficult situations are more likely to be viewed as “challenges to be mastered rather than threats to be avoided” in individuals with high self-efficacy [95]. As previously reported in pediatric cancer context [96,97,98], highly self-efficacious parents may feel relatively comfortable with their ability to cope with the situation, with lower levels of negative affective reactions, included PTSS. Self-compassion, defined as the capacity to assume a warm and understanding attitude toward him/herself in response to negative events or perceived self-inadequacy, was reported as a protective factor in parents [61,98,99]. Better psychological functioning has been related to higher scores in self-compassion [98].

In our review, we found only three studies addressing PTSD in caregivers of children affected by psychiatric disorders, namely one study on parents of children with PTSD [70] and two studies on parents of children with ASD and other rare diseases [69,71]. Although parenting distress has been widely investigated in research studies on caregivers of children affected by ASD [100,101,102,103,104], it seems as the post-traumatic stress burden in caregivers of children and AYAs affected by ASD needs further investigations. Indeed, ASD is a neurodevelopmental disorder that presents an often chronic and disabling clinical picture, characterized by deficits in socio-communicative behaviors, the presence of stereotypical behaviors, and a restricted range of interests [15] but also could be associated to challenging behaviors. The last ones are a range of not socially acceptable conduct that can be physically dangerous to the child or the family, and which have been associated with elevated parenting stress [104,105]. Challenging behaviors that place the child and/or the parent at risk of harm include physical aggression and self-injurious behaviors, suicidal behaviors, elopement, and pica [106,107,108,109]. These behaviors can have a greater physical and emotional impact on parents than core ASD symptoms, bringing a high risk of PTSD [71]. It could be important to identify and treat these subjects with appropriate interventions, as some data report that problem-solving skills training may be beneficial to parents of children with autism spectrum disorder [110].

This review has some limitations that need to be addressed. The first one is the fact that the number of studies was relatively small. In particular, as we described above, only few evince exist on several possible protective or risk factors, and more studies are needed to confirm such results. Furthermore, we found studies on different types of severe diseases, with different prognosis and outcomes, and on different types of young patients (children vs AYAs), that could account for a difference in psychic burden on family caregivers. However, the data reported in literature do not consent a comparison of these categories. Finally, due to the wide heterogeneity of the samples and of the procedures used to assess PTSD and PTSS in the included studies, a meta-analysis process could not be performed.

## 5. Conclusions

Severe illnesses in children and AYAs may represent a complex psychic burden for patients and their caregivers, leading to PTSD and PTSS. Illness related-distress may represent an important risk factor for the development of PTSD symptoms, particularly in subjects with other life stressors and in the presence of other psychiatric symptoms or negative coping styles while facing a great distressing situation, as a severe disease in own’s child. The presence of even a few PTSS in caregivers may compromise their efficacy as caregivers and negatively affect their child’s as well as their own well-being. Identification of risk/protective factors for the development of PTSD may give the opportunity to target particularly at-risk individuals with focused interventions aimed at enhancing potentials for family caregivers to reduce adverse psychic outcomes and to feed psychological well-being in both caregivers and young patients. For this reason, future studies will focus on several relevant risk and protective factors that have not been sufficiently investigated.

## Figures and Tables

**Table 1:** Characteristics of the reviewed studies divided by study type.

Study	Year	Country	Sample	PTSD/PTSS Measures	PTSD/PTSS Rates	Risk/Protective Factors
**Longitudinal studies**						
De Young et al. [60]	2014	Australia	120 parents of children with burn injuries	PDS	PTSD: 22% at 1 month post-injury 5% at 6 months	Risk factors: − Number of invasive procedures − Acute parent distress − Child PTSS − Prior trauma history − Guilt feelings
Harper et al. [45]	2013	USA	75 parents of pediatric cancer patients on the day of each of three medical procedures	IES-R	Not reported	Protective factors: − Task-specific self-efficacy
Malin et al. [56]	2019	USA	162 parents three months after discharge from ≥14 days of NICU	PPQ	PTSD: 25%	Risk factors: − Objective measures of infant illness severity − Parent perception of infant illness
Young et al. [65]	2003	USA	170 caregivers of pediatric transplant recipients	PDS	PTSD: 27.1%	Risk factors: − Negative perceptions − Negative attitudes about their health care services and about health care in general
**Cross-sectional studies**						
Aftyka and Rozalska [55]	2020	Poland	82 parents of infants previously treated in NICU	IES-R	PTSD: 68.5% fathers, 82.9% mothers	Risk factors: − PTSD in the partner − Level of perceived stress (fathers) − Coping styles (emotion-oriented in fathers; avoidance-oriented in mothers)
Bruce et al. [44]	2011	UK	52 parents of children with brain tumor	IES-R	PTSD: 29%	Risk factors: − Number of tumor recurrences Protective factors: − More positive parent-child interaction
Carmassi et al. [57]	2018	Italy	134 Parents of children with a diagnosis of epilepsy	SCID-5 TALS-SR	PTSD: 10.4% parents (13.3% of the mothers and 4.5% of the fathers). Partial PTSD 37.3% (mothers 43.3% and fathers 25.0%)	Risk factors: − Subjective impact of the seizure − Mothers − Manic symptoms (in the fathers)
Casey et al. [69]	2012	USA	265 parents of children with ASD	IES-R	PTSS: 20%	Risk factors: − families with one biological parent not living in the home − families with one or more children diagnosed with ASD − difficulties finding and holding a job − marital problems − depression
Dell’Osso et al. [58]	2018	Italy	134 parents (90 mothers and 44 fathers), of patients aged below 18 years old diagnosed with an epileptic syndrome	SCID-5; TALS-SR	PTSD: 10.4% (13.3% mothers and 4.5% fathers); 37.3% (43.3% of the mothers and 25.0% of the fathers) partial PTSD	Risk factors: − Female − In fathers restricted interests and ruminations
Hawkins et al. [61]	2019	UK	91 parents and primary caregivers (63 mothers, 25 fathers, 3 other) of children during the first 8 weeks following child’s burn injury	IES-R	PTSD: 32.8% of women, 40% of men	Risk factors: − Feelings of guilt and shame − Longer stays in hospital − Younger age of patient Protective factors: − Self-compassion
Ingerski et al. [66]	2010	USA	64 parents of children with chronic illnesses	IES-R	PTSD: 12.9% (14.3% transplantation, 7.7% HIV, 14.3% SCD)	Risk factors: − Transplant candidates
Jurbergs et al. [43]	2009	USA	199 parents of children with cancer	IES-R	Not reported	Risk factors: − Relapsed patients Protective factors: − Being no longer on active treatment
Juth et al. [47]	2015	USA	110 caregivers of AYAs cancer patients	PCL	Not reported	Risk factors: − Illness’s severity perceptions
Khalifa et al. [46]	2014	Egypt	96 parents of children with acute lymphoblastic leukemia and 22 parents of healthy controls. Patients divided into five groups according to disease phase	PTSD Assessment Scale	Not reported	Risk factors: − Mothers Protective factors: − Higher professional and educational levels in parents of children of Group III (during maintenance therapy)
Landolt et al. [41]	2003	Switzerland	355 parents of children 5–6 weeks after an accident or a new diagnosis of cancer or diabetes mellitus type 1	PDS	PTSD: 39.9% (16% fathers and 23.9% mothers)	Risk factors: − Diagnosis of cancer (with respect to other diagnosis) − Poorer functional status of the child
Malpert et al. [48]	2015	USA	127 parents of long-term survivors of childhood Acute Lymphoblastic Leukemia	IES-R	PTSS: 3.9%	Risk factors: − Perceived caregiver strain
Masa’deh and Jarrah [50]	2017	Jordan	416 parents of children with cancer	PCL-C	Not reported	Risk factors: − Mothers − Younger age of parents
McCarthy et al. [49]	2016	Australia	204 parent caregivers of AYAs diagnosed with cancer	PCL-S	PTSS: 42%	Risk factors: − Other life stressors − Impact on plans for the future − Impact on broader family − Living outside the metropolitan area
McCarthy et al. [52]	2018	Australia	204 parent caregivers of AYAs diagnosed with cancer	PCL-S	Not reported	Risk factors: − Higher unmet information needs
Odar et al. [59]	2013	USA	45 parents of children with burn injuries	PCL-S	PTSD: 4.44%	Risk factors: − Younger child age at the time of burn − Greater child PTSS − Family mental health diagnosis − Level of familiar stress prior to the burn event
Pasterski et al. [62]	2014	UK	47 parents of children diagnosed with disorders of sex development	IES-R	PTSD: 49%	Risk factors: − Cognitive confusion − Children diagnosed at earlier age
Perez et al. [53]	2018	USA	59 caregivers of pediatric cancer patients	IES-R	PTSS: 25.42%	Risk factors: − Illness uncertainty − Rumination − Lower income
Perez et al. [63]	2019	USA	139 parents (76 mothers and 63 fathers) of children with disorders of sex development	IES-R	PTSS: 17%	Risk factors: − Mothers − Lower income and increased expenses (in fathers) − Unknown diagnosis (in fathers)
Perez et al. [54]	2020	USA	145 caregivers of children diagnosed with cancer	IES-R	PTSS: 27.4%	Risk factors: − Barriers to care − Illness uncertainty Protective factors: − Support
Phipps et al. [42]	2005	USA	120 parents of children with cancer	IES-R	Not reported	Risk factors: − Mothers − Child PTSS − Recently diagnosed patients
Sawyer et al. [51]	2017	Australia	204 parents of AYAs with cancer	PCL-S	Not reported	Risk factors: − Unmet needs, during and after treatment − Younger age of patient Protective factors: − Meeting support needs
Steinberg et al. [64]	2012	USA	120 caregivers of children with asthma	SCID-I	PTSD: 20.83% (lifetime diagnosis, prior to the past 12-months)	Risk factors: − Beliefs congruent with the lay model of asthma management − Less congruent with the professional model of asthma management
Stewart et al. [71]	2020	Australia	395 parents of children with ASD and rare diseases	PCL-5	PTSD: 40.8% (ASD 23.5%; Rare diseases: 17.3%)	Risk factors: − Challenging child behaviours
Taskiran et al. [68]	2016	Turkey	27 mothers of children who underwent bone marrow transplantation	CAPS	PTSD: 57.6%	Risk factors: − PTSS in children
Tutus & Goldbeck [70]	2016	Germany	113 parents of children and adolescents with PTSD	PDS	PTSS: 48.6%	Risk factors: − Dysfunctional cognitions
Virtue et al. [67]	2014	USA	215 caregivers of children undergoing hematopoietic stem cell transplant (HSCT)	IES-R	PTSS: 54%	Risk factors: − Being married − Depressive and anxiety symptoms − To undergo an allogeneic transplant

IES-r, Impact of Event Scale-Revised; PPQ, Perinatal PTSD Questionnaire; PCL-5, PTSD Checklist for the Diagnostic and Statistical Manual of Mental Disorders 5th edition; PCL-C, PTSD Checklist-Civilian Version; PCL-S, Post-traumatic stress disorder checklist, version S; PDS, Post-traumatic Diagnostic Scale; PTSD, Post-Traumatic Stress Disorder; PTSS, Post-Traumatic Stress Symptoms; SCID-5, Structured Clinical Interview for DSM-5; SCID-I, Clinical Interview for DSM-IV-TR Axis I Disorders; SCID-IV, Structured Clinical Interview for DSM-IV; TALS-SR Trauma and Loss Spectrum—Self Report.

## Data Availability

No new data were created or analyzed in this study. Data sharing is not applicable to this article.
